# The epigenetic signatures of opioid addiction and physical dependence are prevented by D-cysteine ethyl ester and betaine

**DOI:** 10.3389/fphar.2024.1416701

**Published:** 2024-08-30

**Authors:** Jennifer McDonough, Naveen K. Singhal, Paulina M. Getsy, Katherine Knies, Zackery T. Knauss, Devin Mueller, James N. Bates, Derek S. Damron, Stephen J. Lewis

**Affiliations:** ^1^ Department of Biological Sciences, Kent State University, Kent, OH, United States; ^2^ Department of Pediatrics, Case Western Reserve University, Cleveland, OH, United States; ^3^ Department of Anesthesia, University of Iowa Hospitals and Clinics, Iowa City, IA, United States; ^4^ Department of Pharmacology, Case Western Reserve University, Cleveland, OH, United States; ^5^ Functional Electrical Stimulation Center, Case Western Reserve University, Cleveland, OH, United States

**Keywords:** morphine, addiction, dependence, D-cysteine ethyl ester, betaine, human SH-SY5Y cells, rats

## Abstract

We have reported that D,L-thiol esters, including D-cysteine ethyl ester (D-CYSee), are effective at overcoming opioid-induced respiratory depression (OIRD) in rats. Our on-going studies reveal that co-injections of D-CYSee with multi-day morphine injections markedly diminish spontaneous withdrawal that usually occurs after cessation of multiple injections of morphine in rats. Chronically administered opioids are known (1) to alter cellular redox status, thus inducing an oxidative state, and (2) for an overall decrease in DNA methylation, therefore resulting in the transcriptional activation of previously silenced long interspersed elements (LINE-1) retrotransposon genes. The first objective of the present study was to determine whether D-CYSee and the one carbon metabolism with the methyl donor, betaine, would maintain redox control and normal DNA methylation levels in human neuroblastoma cell cultures (SH-SY5Y) under overnight challenge with morphine (100 nM). The second objective was to determine whether D-CYSee and/or betaine could diminish the degree of physical dependence to morphine in male Sprague Dawley rats. Our data showed that overnight treatment with morphine reduced cellular GSH levels, induced mitochondrial damage, decreased global DNA methylation, and increased LINE-1 mRNA expression. These adverse effects by morphine, which diminished the reducing capacity and compromised the maintenance of the membrane potential of SH-SY5Y cells, was prevented by concurrent application of D-CYSee (100 µM) or betaine (300 µM). Furthermore, our data demonstrated that co-injections of D-CYSee (250 μmol/kg, IV) and to a lesser extent, betaine (250 μmol/kg, IV), markedly diminished the development of physical dependence induced by multi-day morphine injections (escalating daily doses of 10–30 mg/kg, IV), as assessed by the lesser number of withdrawal phenomena elicited by the injection of the opioid receptor antagonist, naloxone (1.5 mg/kg, IV). These findings provide evidence that D-CYSee and betaine prevent the appearance of redox alterations and epigenetic signatures commonly seen in neural cells involved in opioid physical dependence/addiction, and lessen development of physical dependence to morphine.

## Introduction

**FIGURE 1:**
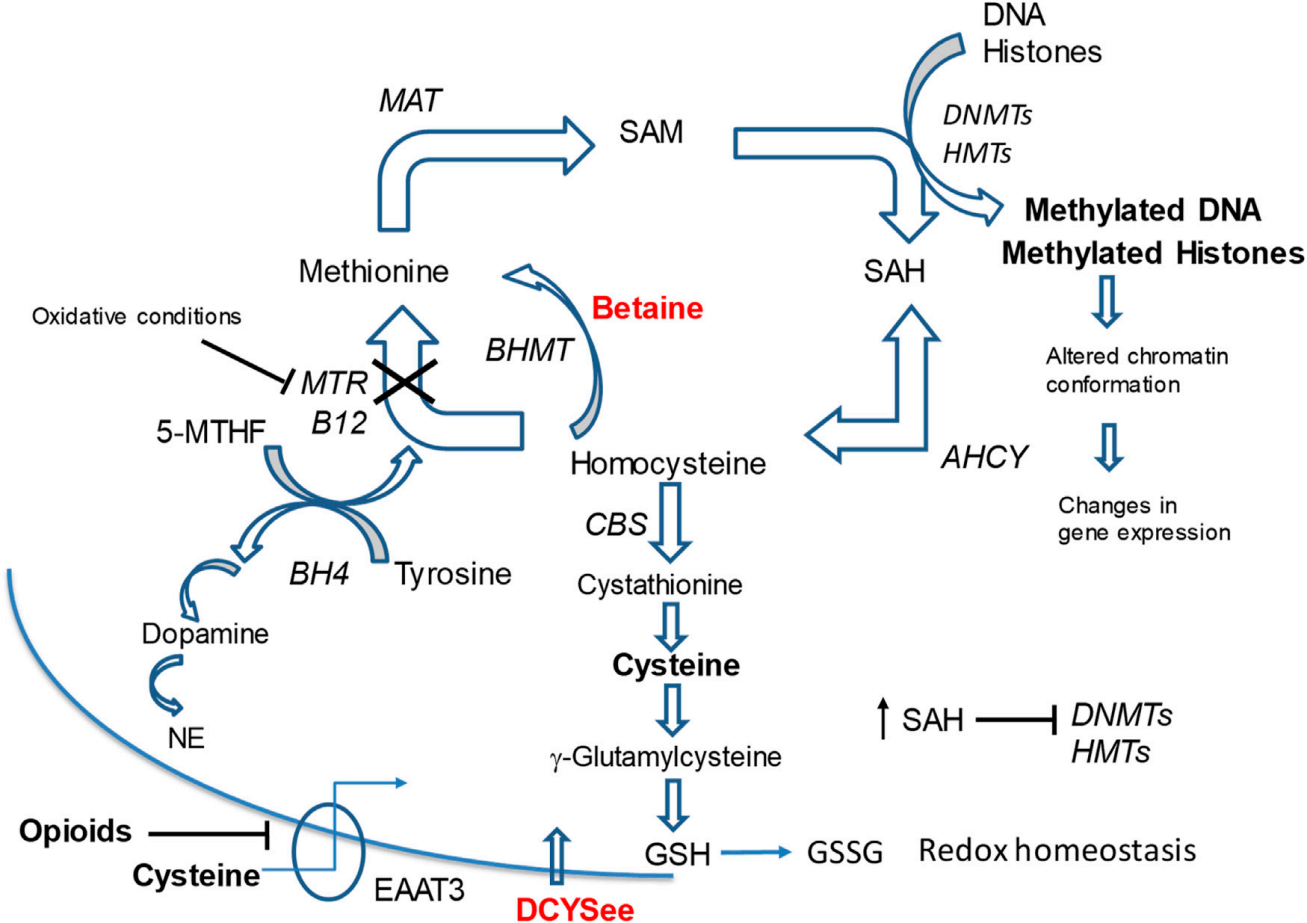
Methionine cycle metabolites and pathways linked to opioid signaling. Neurons depend on transport of L-cysteine by EAAT3 to synthesize the antioxidant glutathione (GSH) since conversion of cystathionine to L-cysteine is limited in neurons. Opioids inhibit EAAT3 causing depletion of L-cysteine and reduced GSH synthesis leading to oxidative stress (decreased GSH/GSSG ratio) and inhibition of MTR. This leads to decreases in SAM levels and a build-up of SAH and homocysteine. The SAM/SAH ratio is decreased, which inhibits activity of DNA DNMTs). This impairs the maintenance of 5-mC status on DNA and alters gene expression programs and cell responses. D-CYSee readily crosses the cell membrane without the EAAT3 transporter and reverses the effects of opioids on DNA methylation. We have evidence that betaine reverses opioid effects on methylation. BHMT bypasses the MTR reaction and drives remethylation of homocysteine to methionine. This process eliminates toxic build-up of homocysteine and SAH in oxidative states so that the SAM/SAH ratio is maintained. Methionine metabolism is linked to neurotransmitter synthesis. The essential cofactor, BH_4_, is synthesized during the methionine synthase reaction, which re-methylates homocysteine to methionine. During this reaction conversion of 5-MTHF to THF creates BH_4_ required for dopamine and NE synthesis. Depletion of cysteine by opioids leads to increased ROS and MTR inhibition, which can reduce BH_4_ availability for synthesis of neurotransmitters dopamine and NE. Abbreviations: MAT, methionine adenosyltransferase (S-Adenosylmethionine synthetase); SAM, S-adenosylmethionine; SAH, S-adenosyl-homocysteine; DNMT, DNA methyl-transferases; HMT, histone methyltransferases; BHMT, betaine homocysteine methyl-transferase; MTR, methionine synthase; B12, Vitamin B12; CBS, cystathione β synthase; AHCY, S-adenosylhomocysteine hydrolase; 5-MTHF, L-methyl-folate; BH4, tetrahydrobipterin; GSH, glutathione; 5-mC, 5-methylcytosine; GSSG, glutathione dipeptide (oxidized); NE, norepinephrine; EEAT3, excitatory amino acid transporter-3; D-CYSee, D-cysteine ethyl ester.

Opioids alter DNA and histone methylation patterns, and gene expression, which contributes to their physiological action and the underlying addictive nature of these drugs (Trivedi et al., 2014a; Trivedi et al., 2014b; Trivedi and Deth, 2015; De Sa Nogueira et al., 2018). Methylation of DNA and histones can cause heritable epigenetic changes in chromatin structure that activate or silence gene transcription. While some epigenetic marks are stable, others are more dynamic allowing cells to respond rapidly to changing metabolic or environmental signals (Suzuki and Bird, 2008; Ma et al., 2009; Ito et al., 2010; Gut and Verdin, 2013; Smith and Meissner, 2013; Salminen et al., 2014). The mechanisms associated with opioid-induced epigenetic changes involve inhibition of activity and ultimate degradation of the excitatory amino acid transporter 3 (EAAT3), which is required for uptake of the amino acid, L-cysteine, into neurons (Trivedi et al., 2014a; Trivedi et al., 2014b; Trivedi and Deth, 2015; De Sa Nogueira et al., 2018). L-cysteine exerts a variety of effects in cells, including conversion to the antioxidant tripeptide, glutathione (GSH, γ-glutamyl-cysteinyl-glycine), which maintains redox homeostasis in cells (Lu, 2013; Jenkins et al., 2021). Neurons are particularly sensitive to L-cysteine deficiency because flux, via transulfuration pathways, which convert L-homocysteine to GSH, are extremely limited due to low conversion of L-cystathionine to L-cysteine. Neurons rely on L-cysteine transport from astrocytes via the EAAT3 transporter (Trivedi and Deth, 2015; Trivedi et al., 2015). As a direct consequence of opioid exposure (Figure 1), neurons become deficient in L-cysteine and GSH, which results in an inability to maintain redox control (Trivedi et al., 2014a; Trivedi et al., 2014b; Trivedi and Deth, 2015; Trivedi et al., 2015).

Impaired redox control in cells plays an important role in the expression of opioid withdrawal signs (Xu et al., 2006), thus therapeutics that maintain redox homeostasis (e.g., GSH levels) may be beneficial for treating opioid use disorder (OUD). Redox control and DNA/histone methylation reactions are closely linked to intracellular methionine metabolism (Figure 1). In the methionine cycle, methionine is converted to S-adenosyl-methionine (SAM), which is the methyl donor for most methylation reactions (e.g., DNA and histone methylation) within cells (Bottiglieri, 2013; Froese et al., 2019). The B_12_-dependent methionine synthase (MTR in Figure 1) is sensitive to inhibition by reactive oxygen species (ROS) via the oxidation of B_12_ (cobalamin) (Nicolaou et al., 1994; Nicolaou et al., 1996; Mukherjee and Brasch (2011). With opioid exposure, the oxidation of MTR contributes to decreased levels of the methyl donor SAM for the epigenetic regulation of chromatin (Trivedi and Deth, 2015; Trivedi et al., 2015). SAM is converted to S-adenosyl-homocysteine (SAH) after donating a methyl group. Under oxidative-stress states, the build-up SAH and homocysteine, inhibits the activities of methyltransferase enzymes (Cantoni, 1975; Xu et al., 2015). The perturbation of redox control and SAM/SAH ratio (i.e., methylation potential), could readily explain the reduction in DNA and histone methylation by opioids (Sun et al., 2012; Trivedi et al., 2014a; Trivedi et al., 2014b). Changes in the SAM/SAH ratio lead to changes in the methylation status of downstream substrates, including DNA and histones, and results in aberrant gene expression (Mentch et al., 2015). Morphine treatment of human neuroblastoma cells causes overall decreases in DNA methylation, which results in the transcriptional activation of previously silenced long interspersed elements (LINE-1) retrotransposon genes (Trivedi et al., 2014a; Trivedi et al., 2014b). It should be noted that opioids also alter the expression of genes involved in neurotransmission, synaptic plasticity, and GSH metabolism (McClung et al., 2005; Tapocik et al., 2013).

**FIGURE 2:**
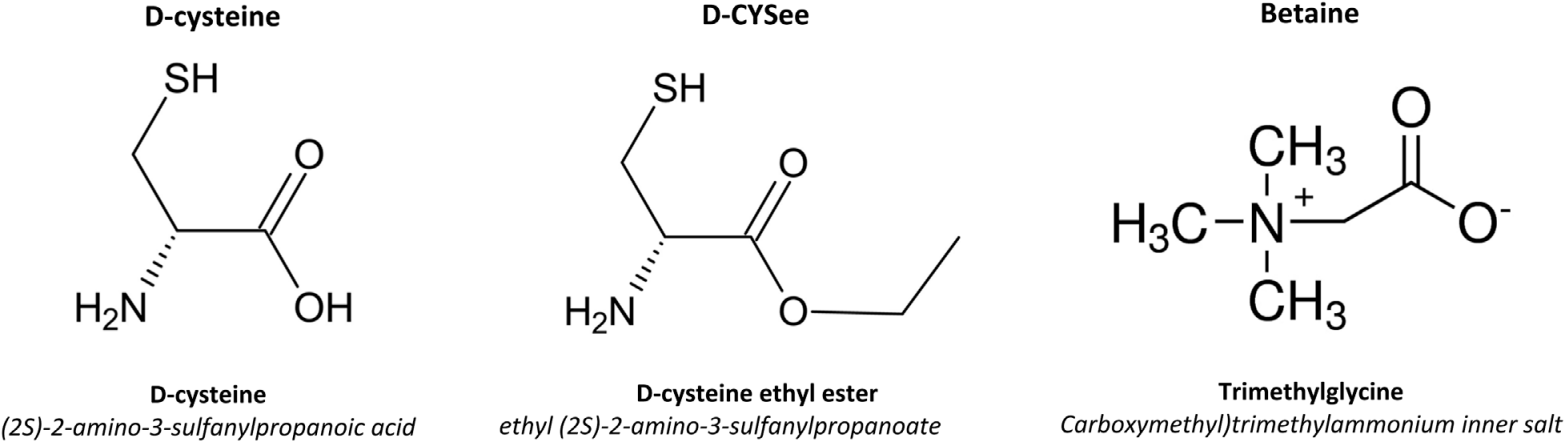
Chemical structures and names of D-cysteine, D-cysteine ethyl ester (D-CYSee) and betaine.

**FIGURE 3:**
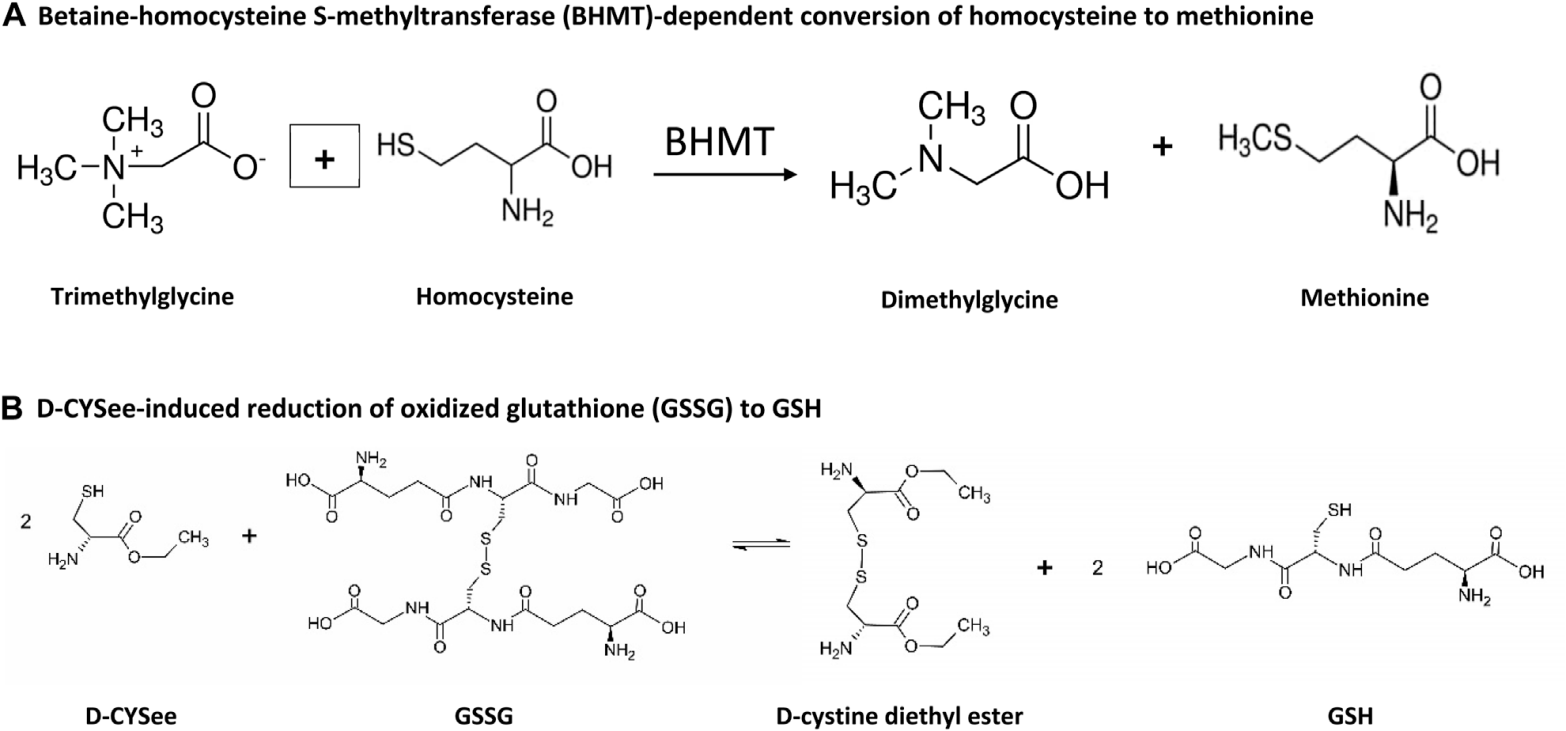
Steps by which betaine and D-CYSee may contribute to the alteration of methionine and GSH status. **(A)** Betaine-homocysteine S-methyltransferase (BHMT)-dependent conversion of homocysteine to methionine. **(B)** D-CYSee-induced reduction of oxidized glutathione (GSSG) to GSH.

We have reported that L-cysteine ethyl ester (L-CYSee), and related L,D-thiolesters (Mendoza et al., 2013; Gaston et al., 2021; Jenkins et al., 2021; Getsy et al., 2022a; Getsy et al., 2022b; Lewis et al., 2022) and L-S-nitrosthiols (Getsy et al., 2022c; Getsy et al., 2022d), overcome the deletrious actions of fentanyl and morphine on breathing, arterial blood-chemistry and alveolar gas exchange in rats, while not markedly affecting the analgesic/sedative actions of the opioids. We have also demonstrated that D-cysteine ethyl ester (D-CYSee) mimics the effects of L-CYSee and chose to use this compound because it is likley to have less off-target effects than L-CYSee (Getsy et al., 2022c; Getsy et al., 2022d). Although we have not determined the mechanisms by which D-CYSee or other D,L-thiolesters reverse opioid-induced respiratory depression (OIRD), we reported that the free radical and superoxide anion scavenger, Tempol, also blunted fentanyl- and morphine-induced OIRD (Baby et al., 2021a; Baby et al., 2021b). As such, it is possible that preventing/reversing opioid-induced changes in oxidation-reduction status of neurons may be of benefit in overcoming OIRD and physical dependence to opioids. In this study, our first objective was to test whether enhancing redox control with D-CYSee and the one-carbon metabolite with a methyl donor, betaine (Ueland et al., 2005; Chen and Murata, 2008; Lever and Slow, 2010; Ueland, 2011; Aramburu et al., 2014; Kumar et al., 2016; Knight et al., 2017; Zhao et al., 2010; Ohnishi et al., 2019; Arumugam et al., 2021) (Figure 2 for chemical structures), would maintain redox control and DNA methylation levels in human SH-SH5Y neuroblastoma cells (Singhal et al., 2015; Brown et al., 2016) that were challenged overnight with morphine (see Figure 3 for potential steps). We chose to study human SH-SH5Y neuroblastoma cells to allow comparisons to the extensive findings about redox-dependent pathways in these cells (Núñez et al., 2004; Tirmenstein et al., 2005; Aguirre et al., 2007; Dias et al., 2014; Wang et al., 2019; Mistry et al., 2020), and the mechanisms of action of agents that modify the cell signaling effects of opioids in these cells (Trivedi et al., 2014a; Trivedi et al., 2014b; Trivedi et al., 2015; Trivedi and Deth, 2015).

The second objective was to determine whether intravenous co-injections of D-CYSee or betaine modified the development of physical dependence to morphine in male Sprague Dawley rats as assessed by the strength of the withdrawal responses elicited by the injection of the opioid receptor antagonist, naloxone HCl (NLX). The behavioral phenomena included the occurrence of jumping (all four paws off the floor), wet dog-like shakes (WDS), rearing on hind legs (rears), episodes of fore-paw licking (FPL), circling (full 360° rotation), writhing (fully body contortion) and sneezing (abrupt expulsion of air). The other recorded parameters were drops in body temperature and body weight. All responses are classic signs of NLX-induced responses in morphine-dependent rats (Laska and Fennessy, 1976; Hutchinson et al., 2007; Morgan and Christie, 2011; Nielsen and Kreek, 2012).

## Materials and methods

### Permissions, rats, and surgical procedures

All studies were carried out in strict accordance with the NIH Guide for Care and Use of Laboratory Animals (NIH Publication No. 80-23) revised in 1996, and in strict compliance with the ARRIVE (Animal Research: Reporting of *In Vivo* Experiments) guidelines (http://www.nc3rs.org.uk/page. asp? id = 1,357). All protocols involving the use of rats were approved by Animal Care and Use Committees of Kent State University and Case Western Reserve University. Adult male Sprague Dawley rats were purchased from *ENVIGO* (Madison, WI, USA). They were given 5 days to recover from transportation before surgery. All rats received an indwelling jugular vein catheter (PE-10 connected to PE-50) under 2%–3% isoflurane anesthesia as described previously (Gaston et al., 2021; Getsy et al., 2022f). The catheter was then exteriorized at the back of the neck and all wounds were closed. The rats were given 3 days to recover from the surgeries. The venous catheters were flushed with 0.3 mL of phosphate-buffered saline (0.1 M, pH 7.4) 3–4 h before commencement of each of the studies. All studies were done in a quiet room with relative humidity of 50% ± 2% and temperature of 21.3°C ± 0.2°C. (+)-Morphine sulfate was obtained from *Baxter Healthcare* (Deerfield, IL, USA). D-CYSee HCl powder was obtained from *ChemImpex* (Wood Dale, IL). D-cysteine, betaine, and naloxone HCl were obtained from *Sigma-Aldrich* (St. Louis, MO, USA). Full step by step instructions with detailed diagrams for the above surgeries and vascular catherizations can be found at https://www.criver.com/products-services/research-models-services/preconditioning-services/rodent-surgery/vascular-catheterizations?region=3601.

### Injection protocols

All co-injections started at 8 a.m. and 8 p.m. except for the final injection which was given at 2 p.m. Each group consisted of nine adult male Sprague Dawley rats. Day 1: two injections of morphine (10 mg/kg, IV). Day 2: two injections of morphine (15 mg/kg,IV). Day 3: two injections of morphine (20 mg/kg, IV). Day 4: two injections of morphine (25 mg/kg,IV). Day 5: two injections of morphine (30 mg/kg, IV). One group of rats received injection of vehicle (saline) either 15 min (n = 5) or 5 min (n = 4) prior to the injection of morphine. Other groups of rats (n = 9 rats per group) received co-injections of a 250 μmol/kg dose of (a) betaine (29.3 mg/kg, IV), (b) D-cysteine (30.3 mg/kg, IV), or (c) D-CYSee (46.5 mg/kg, IV) with morphine. Rats were placed in clear plastic boxes immediately after the last set of co-injections and allowed 60 min to acclimatize. At 60 min, all rats received an injection of NLX (1.5 mg/kg, IV) and the NLX-precipitated withdrawal phenomena were recorded over a 90 min period by three observers who were blind to the drug-administration protocols.

### Tissue culture and redox levels

Human SH-SH5Y neuroblastoma cell lines were maintained in DMEM/F12 (*Sigma-Aldrich, St Louis, MO*) supplemented with 10% fetal bovine serum (*Atlanta Biologicals, Atlanta, GA*), 50 μg/mL penicillin, and 50 μg/mL streptomycin (*Corning*, Corning, NY) at 37°C in a humidified CO_2_ incubator. Cells were grown in 10 cm Petri dishes in confluence up to 90%. Cells were treated overnight with morphine (100 nM), D-CYSee (100 µM), betaine (300 µM), morphine (100 nM) + D-CYSee (100 µM) or morphine (100 nM) + betaine (300 µM). GSH levels (ng/mL) were measured with a GSH kit (*Abcam, Cambridge, United Kingdom*) according to manufacturer’s instructions from 4 separate experiments. A standard curve was constructed to determine actual GSH levels with absorbance units at each concentration being 0 (0 ng/mL), 0.242 (200 ng/mL), 0.549 (400 ng/mL), 1.207 (600 ng/mL), 1.666 (800 ng/mL) and 1.837 (1,000 ng/mL). Mitochondrial membrane potential was measured in human SH-SY5Y neuroblastoma cells by measuring JC-1 (5,50,6,60-tetra-chloro-1,10,3,30-tetraethylbenzimidazolylcarbocyanine iodide) fluorescence as described by Chen et al. (2019). In the normal cells, JC-1 exists as a monomer in the cytosol (green) and also accumulates as aggregates (red) in mitochondria induced by higher mitochondrial membrane potential. In apoptotic and necrotic cells, JC-1 exists in monomeric form and stains the cytosol green. As such, the red fluorescence denotes healthy mitochondria with intact membrane potential, whereas the green fluorescence denotes damaged mitochondria with altered membrane potential. Quantitation of red and green fluorescence and red/green fluorescence ratio denoting healthy/damaged mitochondria was measured in at least 3 separate treatments using a Tecan Safire5 microplate reader.

### DNA methylation studies

Human SH-SY5Y neuroblastoma cells were treated overnight (20 h) with morphine (100 nM), D-CYSee (100 μM), betaine (300 μM), morphine (100 nM) + D-CYSee (100 μM) or morphine (100 nM) + betaine (300 μM). Global DNA methylation levels were measured (5-methylcytosine (5-mC)/100 ng DNA) with *MethylFlash Methylated DNA Quantification Kits* (*Epigentek*, Farmingdale, NY) from at least 3 separate treatments.

### Real-time quantitative reverse transcription polymerase chain reaction (qRT-PCR)

Levels of LINE-1 RNA were measured by qRT-PCR with gene specific primers as described by Trivedi et al. (2014a, 2014b). Total RNA was isolated from SH-SY5Y cells treated overnight (20 h) with morphine (100 nM), D-CYSee (100 μM, betaine (300 μM), morphine (100 nM) + D-CYSee (100 μM) or morphine (100 nM) + betaine (300 μM) using the TRIzol reagent (*Thermo Fischer Scientific*, Waltham, MA). The samples were t purified on *Quick-RNA MiniPrep Plus kit* columns (*Zymo Research*, Irvine, CA). qRT-PCR was performed in triplicate with *Brilliant III Ultra-Fast SYBR-Green* (*Agilent Technologies*, Santa Clara, CA) and a *MaxPro3000 Real Time PCR* system (*Agilent Technologies*, Santa Clara, CA). Data was collected from 3 separate cell preparations. Relative gene expression was calculated with the 2^−ΔΔCt^ method after normalization to β-actin levels.

### Data analyses

All data were analyzed using one-way and two-way ANOVA followed by Bonferroni corrections for multiple comparisons between means using the error mean square terms from each ANOVA analysis (Wallenstein et al., 1980; Ludbrook, 1998; McHugh, 2011) as detailed previously (Getsy et al., 2023a; Getsy et al., 2023b). A *p* < 0.05 value denoted the initial level of statistical significance that was modified according to the number of comparisons between means as described by Wallenstein et al. (1980). The modified *t-*statistic is t = (mean group 1 - mean group 2)/[s x (1/n_1_ + 1/n_2_)^1/2^] where s^2^ = the mean square within groups term from the ANOVA (the square root of this value is used in the modified *t*-statistic formula) and n_1_ and n_2_ are the number of rats in each group under comparison. Based on an elementary inequality called Bonferroni’s inequality, a conservative critical value for modified *t*-statistics obtained from tables of *t*-distribution using a significance level of P/m, where m is the number of comparisons between groups to be performed (Winer, 1971). The degrees of freedom are those for the mean square for within group variation from the ANOVA table. The critical Bonferroni value is not found in conventional tables of the *t*-distribution, but can be approximated from tables of the normal curve by t = z + (z + z^3^)/4n, with n being the degrees of freedom and z being the critical normal curve value for P/m. The Bonferroni procedure provides critical values that are lower than those of other procedures when the number of comparisons can be limited, and will be slightly larger than those of other procedures if many comparisons are made (Wallenstein et al., 1980). Statistical analyses were performed with the aid of GraphPad Prism software (*GraphPad Software*, Inc., La Jolla, CA). All summary data are presented as mean ± SEM.

## Results

### Effects of D-CYSee and betaine on GSH levels and mitochondria after opioid exposure

**FIGURE 4:**
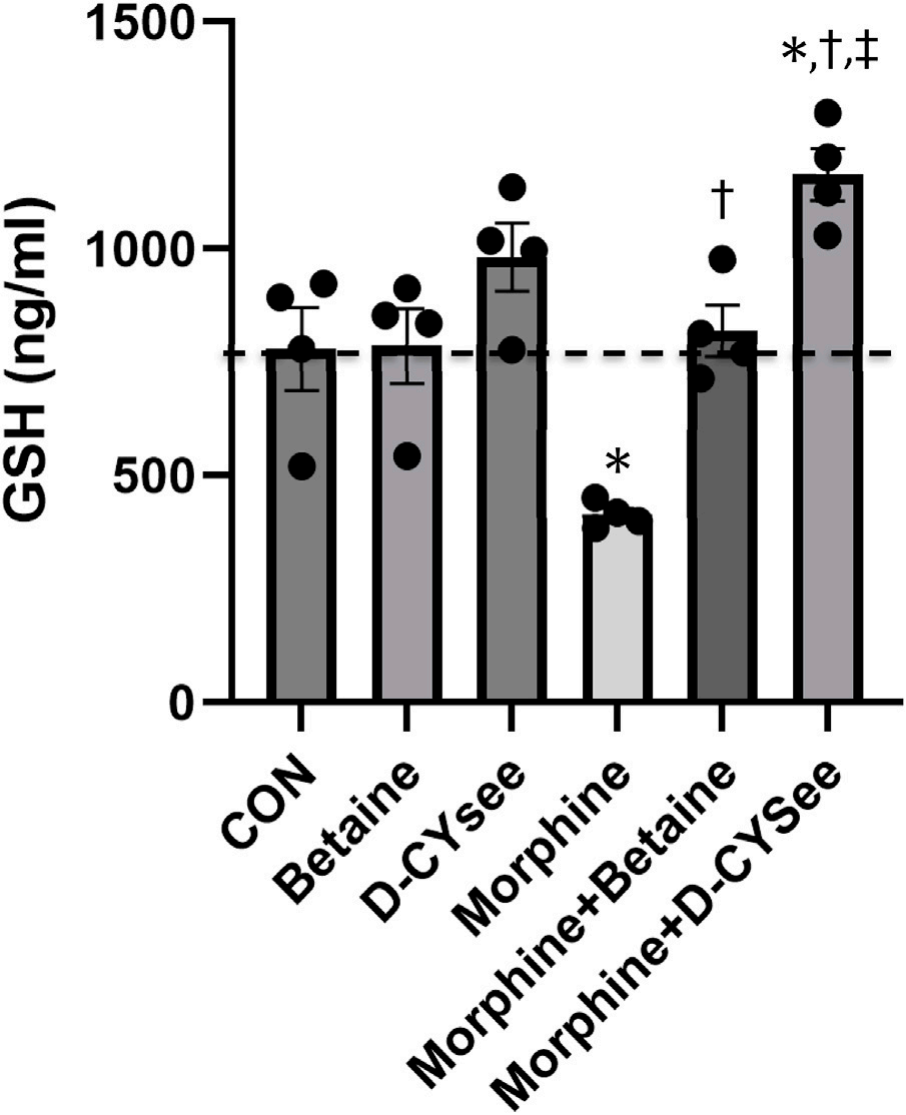
D-CYSee reverses morphine effects on cellular antioxidant capacity. GSH concentrations were measured in human SH-SY5Y human neuroblastoma cells. Treatment groups: CON, control; betaine (300 μM); D-cysteine ethyl ester (D-CYSee, 100 μM); morphine (100 nM); morphine (100 nM) + betaine (300 μM); Morphine (100 nM) + D-CYSee (100 μM); There were four cell preparations in each group. Data are presented as mean ± SEM. *p < 0.05, significantly different to control. ^†^ *p* < 0.05, morphine + betaine or D-CYSee *versus* morphine alone.

**FIGURE 5:**
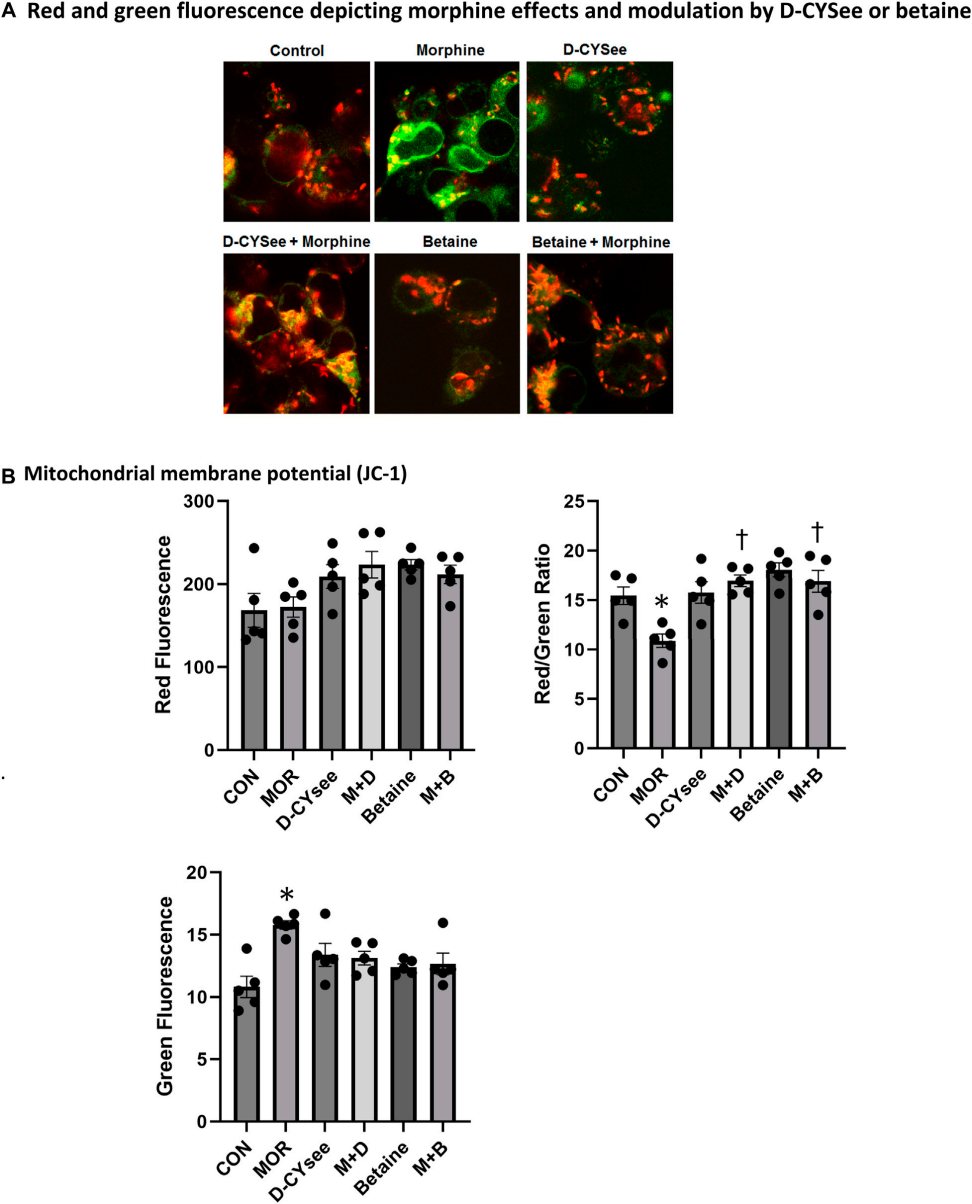
Morphine damage to mitochondria is rescued by D-CYSee or betaine. (Panel A) JC-1 staining of mitochondria (red). Damaged mitochondria lose membrane potential, indicated by reduced red and increased green fluorescence. (Panel B) Quantitation is shown for overall red, green and red/green fluorescence. CON, control. MOR, morphine; M + D, morphine + D-CYSee; M + B, morphine + betaine. *p < 0.05, significant difference from control levels. ^†^ *p* < 0.05, morphine + treatment *versus* morphine alone.

Opioids decrease L-cysteine uptake, resulting in decreased levels of reduced glutathione (GSH) that leave neurons vulnerable to oxidative insults. As summarized in Figure 4, overnight incubation with betaine (300 μM) or D-CYSee (100 μM) did not alter GSH levels in human SH-SY5Y cells, whereas morphine (100 nM) produced a substantial decrease in GSH levels. Co-incubation with betaine (300 μM) prevented morphine (100 nM) from decreasing GSH levels. D-CYSee (100 μM) also overcame the ability of morphine (100 nM) to depress GSH levels. In fact, GSH levels were actually higher than control levels in cells co-incubated with morphine and D-CYSee. We also tested the effects of D-CYSee and betaine on mitochondrial membrane potential in human SH-SY5Y neuroblastoma cells by measuring JC-1 fluorescence. Morphine reduced membrane potential by 30% in neuroblastoma cells. D-CYSee and betaine were equally effective in restoring mitochondrial membrane potential during morphine treatment (Figure 5). As such, it is evident that D-CYSee and betaine maintain redox homeostasis and protect mitochondria from opioid-induced toxicity.

### D-CYSee and betaine reverse opioid mediated reductions in global levels of DNA methylation and restore appropriate gene silencing

**FIGURE 6:**
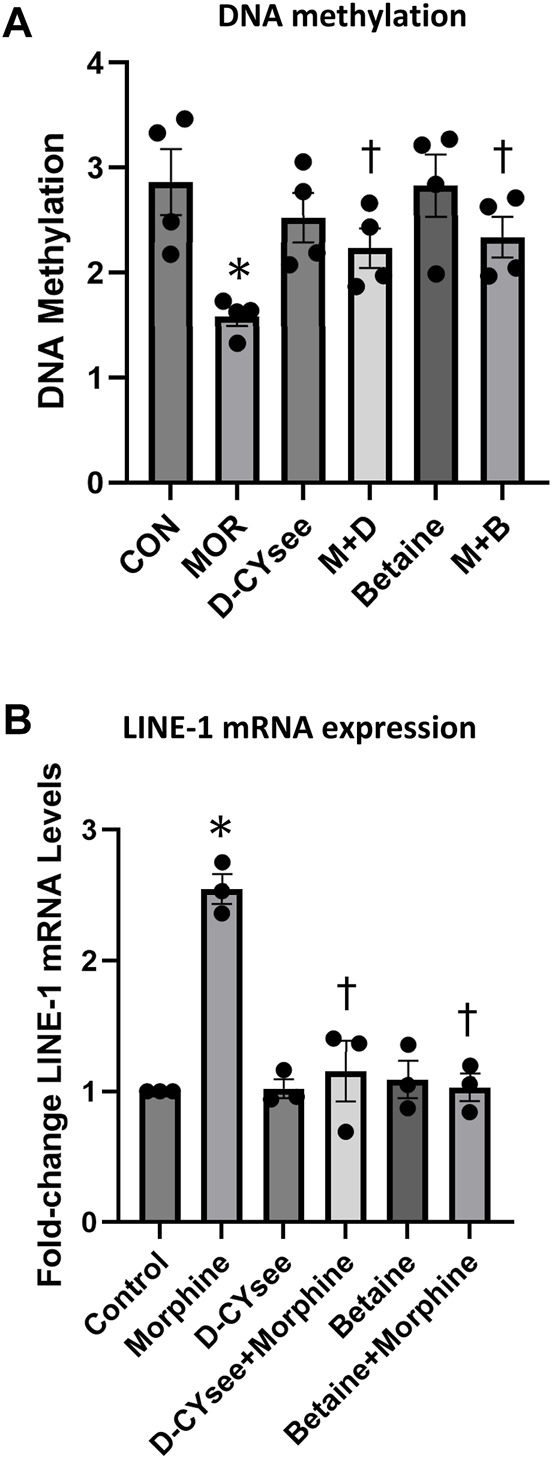
D-CYSee or betaine reverses the effects of morphine on global DNA methylation (Panel A) and LINE-1 RNA expression (Panel B) in human SH-SY5Y neuroblastoma cells. The cells were treated overnight (16 h) with morphine (0.1 μM) in the absence or presence of D-CYSee (100 μM) or betaine (300 μM). Abbreviations: MOR, morphine. M + D, morphine + D-CYSee. M + B, morphine + betaine. There were 3 separate samples in each group. *p < 0.05, significantly different from control. ^†^ *p* < 0.05, morphine + treatment *versus* morphine alone.

We demonstrate that D-CYSee and betaine reverse morphine-induced changes in global DNA methylation. Human SH-SY5Y neuroblastoma cells were treated with 100 nM morphine overnight and, as seen in Figure 6A, morphine reduced global DNA methylation. Methylation levels were restored in cells co-treated overnight with D-CYSee (100 μM) or betaine (300 μM). We then determined the effects of D-CYSee and betaine on morphine-induced changes in LINE-1 RNA expression. To determine whether the reductions in 5-mC shown in Figure 6A, which silences transcription, effected the expression of the LINE-1 gene, we measured levels of LINE-1 RNA by qRT-PCR. As seen in Figure 6B, LINE-1 RNA levels were increased by over 2-fold with morphine treatment and these RNA levels were restored to control levels with D-CYSee or betaine.

### D-CYSee and betaine diminish the development of physical dependence to morphine

**FIGURE 7:**
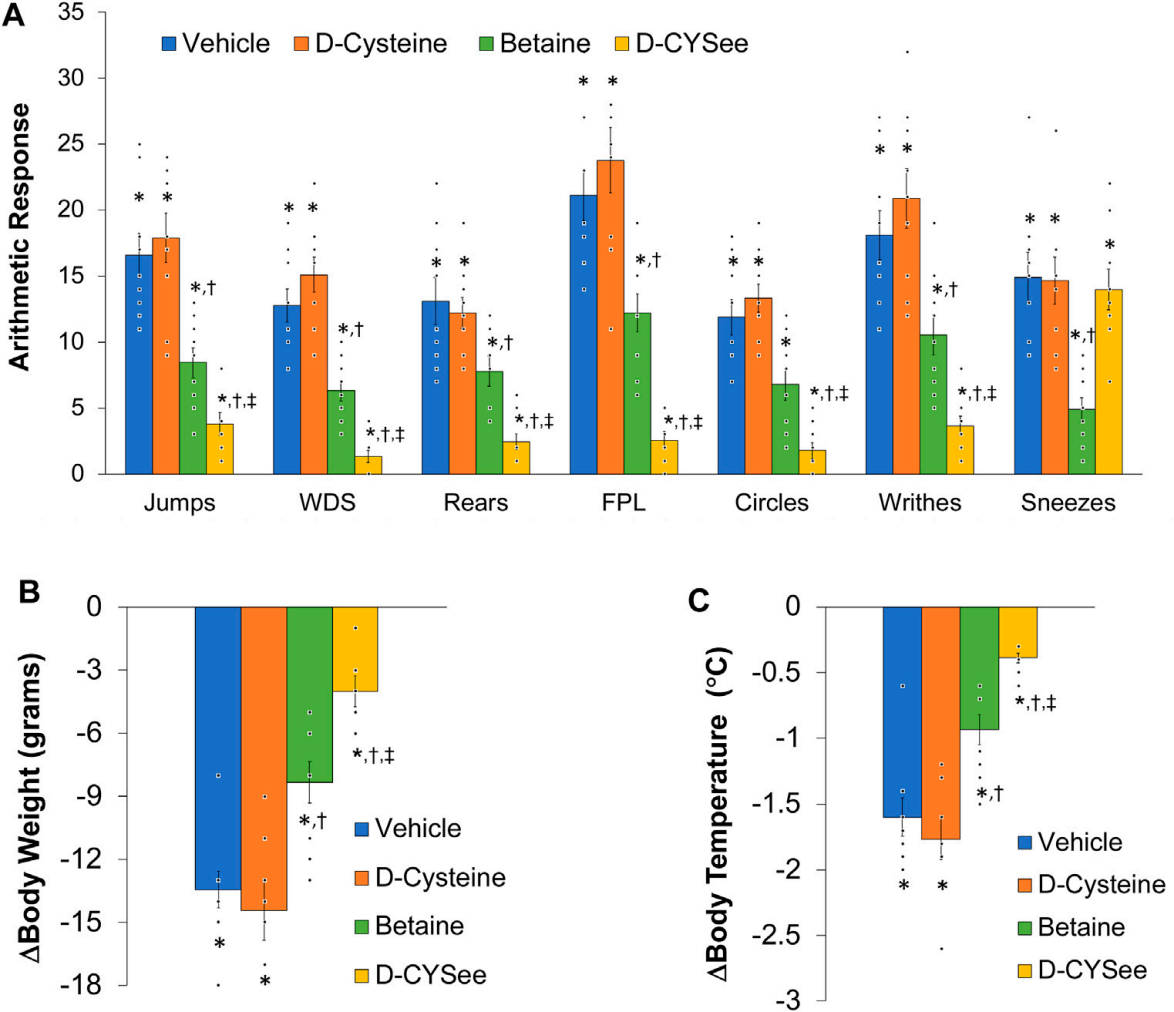
Panel **(A)** Withdrawal phenomena elicited by a bolus injection of naloxone HCl (1.5 mg/kg, IV) in rats that received co-injections of morphine (doses escalating from 10 mg/kg to 30 mg/kg, IV) + vehicle (saline), D-cysteine (250 μmol/kg, IV), betaine (250 μmol/kg, IV) or D-cysteine ethyl ester (D-CYSee, 250 μmol/kg, IV). Withdrawal Signs: Jumps, all four paws off the floor; WDS, wet-dog shakes; Rears, rearing on hind legs; FPL, episodes of fore-paw licking; Circles, a 360° rotation; Writhes, fully body contortion; Sneezes, abrupt expulsion of air. Panel **(B)** Changes in body weight elicited by injection of naloxone HCl (1.5 mg/kg, IV) in the above groups of rats. Panel **(C)** Changes in body temperature elicited by injection of naloxone HCl (1.5 mg/kg, IV) in the above groups of rats. The data are mean ± SEM. There were nine rats in each group. *p < 0.05, significant response from Pre-values. ^†^ *p* < 0.05, D-cysteine, D-CYSee or betaine *versus* vehicle. ^‡^ *p* < 0.05, D-CYSee v*ersus* betaine.

NLX elicited a pronounced series of behaviors (Figure 7A) and falls in body weight (Figure 7B) and body temperature (Figure 7C) in rats that received co-injections of morphine + vehicle. These NLX-induced responses were reduced in rats that received co-injections of betaine and markedly reduced in rats co-injected with D-CYSee, but not D-cysteine. As seen in Table 1, body temperature was equally elevated 60 min after the last set of co-injections of morphine + vehicle or morphine + D-cysteine (Pre-NLX). This hyperthermia was less in rats co-injected with morphine + betaine and markedly reduced in those co-injected with morphine + D-CYSee. As also seen in Table 1, body weight was equally reduced 60 min after the last set of co-injections of morphine + vehicle or morphine + D-cysteine (Pre-NLX). There was no reduction in the body weights of rats co-injected with morphine + betaine and morphine + D-CYSee, instead an increase in body weight was observed at this timepoint (Pre-NLX) compared to their starting weights (Pre values). NLX elicited pronounced falls in body weights in the morphine + vehicle and morphine + D-cysteine treatment groups. The NLX-induced falls in body weight were less in the rats that received morphine + betaine, and markedly less in the rats that received morphine + D-CYSee.

**TABLE 1:** Changes in body temperature and body weight elicited by the injection of naloxone HCl in the four treatment groups.

Body temperature (°C)			Actual values			ΔChange (°C)	
Emulsion	Infusion	Injection	Pre	Pre-NLX	Post-NLX	Pre-NLX vs. pre	NLX response
Morphine	Vehicle	NLX	37.4 ± 0.1	38.4 ± 0.1	36.8 ± 0.2	+0.96 ± 0.10*	−1.63 ± 0.15*
Morphine	D-cysteine	NLX	37.5 ± 0.1	38.5 ± 0.2	36.8 ± 0.3	+1.04 ± 0.08^*^	−1.77 ± 0.16*
Morphine	D-CYSee	NLX	37.5 ± 0.1	37.8 ± 0.1	37.4 ± 0.2	+0.26 ± 0.06*^,†^	−0.39 ± 0.04*^,†^
Morphine	Betaine	NLX	37.5 ± 0.1	38.1 ± 0.1	37.2 ± 0.1	+0.58 ± 0.06*^,†,‡^	−0.93 ± 0.11*^,†,‡^

Body Weight (grams)			Actual Values			ΔChange (grams)	
Emulsion	Infusion	Injection	Pre	Pre-NLX	Post-NLX	Pre-NLX vs. Pre	NLX response
Morphine	Vehicle	NLX	331 ± 2.1	320 ± 2.0	307 ± 1.8	−10.8 ± 1.1*	−13.4 ± 0.9*
Morphine	D-Cysteine	NLX	334 ± 1.6	323 ± 1.3	308 ± 2.1	−11.1 ± 0.7*	−14.4 ± 1.4*
Morphine	D-CYSee	NLX	333 ± 1.7	342 ± 2.0	338 ± 2.0	+9.2 ± 1.0*^,†^	−4.0 ± 0.7*^,†^
Morphine	Betaine	NLX	334 ± 1.4	336 ± 1.7	327 ± 2.3	+2.1 ± 0.8^†,‡^	−8.3 ± 1.1*^,†,‡^

Responses elicited by the acute injection of naloxone HCl (NLX, 1.5 mg/kg, IP) in rats that received co-injections of morphine plus vehicle (300 μL/kg, IV), D-cysteine, D-cysteine ethyl ester (D-CYSee), or betaine (250 μmol/kg, IV). There were nine rats in each group. The data are presented as mean ± SEM.

*p < 0.05, significant response from Pre-values.

^†^ *p* < 0.05, D-cysteine, D-CYSee, or betaine *versus* vehicle.

^‡^ *p* < 0.05, betaine *versus* D-CYSee.

## Discussion

Trivedi et al. (2014a, 2014b) provided compelling evidence that morphine induced dependence/addiction involves redox-based changes in global DNA methylation and retrotransposon transcription via the inhibition of excitatory amino acid transporter type 3 (EAA3)-mediated uptake of cysteine into brain neurons. Some steps arising from studies of Trivedi et al. (2014a), Trivedi et al. (2014b) and others (Lin et al., 2001; Ikemoto et al., 2002; Mao et al., 2002; Xu et al., 2003; 2006; Christie, 2008; Yang et al., 2008; Wang et al., 2009; Daijo et al., 2011 Gutowicz et al., 2011; Liu et al., 2011; Maze and Nestler, 2011; Lim et al., 2012; Sun et al., 2012; Browne et al., 2020) appear to involve (1) morphine attenuation of L-cysteine uptake into neurons by G-protein-dependent decrease in EAA3 expression and function, (2) reductions in intracellular levels of L-cysteine, L-glutathione and methylation index (SAM/SAH ratio, S-adenosyl-methionine/S-adenosyl-homocysteine), (3) reduced methylation of global CpG (regions of DNA in which a cytosine nucleotide is followed by a guanine nucleotide in linear base sequence along the 5′→ 3′direction), and decreased CpG methylation of long interspersed nuclear element - 1 (LINE-1) retrotransposon regulatory regions, (4) activation of transcription of previously silenced LINE-1 gene [see Figure 5 of Trivedi et al. (2014a)]. It occurred to us that co-administration of cell-penetrant L,D-thiolesters, such as L- or D-CYSee or the methyl donor, betaine, may be able to prevent the redox changes associated with a perturbation of DNA methylation and gene expression in neurons.

The present study found that overnight exposure to morphine greatly reduced intracellular levels of GSH in human SH-SY5Y neuroblastoma cells. These findings are consistant with substantial evidence that opioids/opioid peptides decrease the levels of GSH in neuronal, non-neuronal cells and organelles by a number of mechanisms (Moussa and el-Beih, 1972; Eklöw-Låstbom et al., 1986; Skoulis et al., 1989; Goudas et al., 1999; Guzmán et al., 2009; Deb and Das, 2011; Abdel-Zaher et al., 2013a; Abdel-Zaher et al., 2013b; Trivedi et al., 2014a; Trivedi et al., 2014b, Trivedi et al., 2015; Trivedi et al., 2016; Trivedi and Deth, 2015; Samikkannu et al., 2015; Yun et al., 2015; Yun et al., 2017; Chen et al., 2019; Shibani et al., 2019; Osmanlıoğlu et al., 2020; Mozafari et al., 2022), including decreased glutathione synthase activity (Samikkannu et al., 2015), increased glutathione peroxidase activity (Mozafari et al., 2022), and formation of glutathione conjugates, such as *formyl glutathione* (Eklöw-Låstbom et al., 1986). GSH is present in all mammalian cells and is vital to cell health (Townsend et al., 2003; Dwivedi et al., 2020). The presence of a sulfhydryl (SH) group confers potent antioxidant efficacy to GSH by, for example, its interactions with reactive oxygen and nitrogen species (Keszler et al., 2010; Mailloux et al., 2013) in which two molecules of GSH dimerize via disulfide linkage to form GSSG (glutathione disulfide) Mailloux et al., 2013. The reduced (GSH) and oxidized disulfide form (GSSG) are readily inter-convertible, with reduced GSH being the predominant form in healthy cells (Lu, 2013). GSH acts in coordination with other redox-active agents, such as α-lipoic acid and nicotinamide adenosine diphosphate (NADPH), to regulate intra-cellular redox status (Shen et al., 2005). GSH is converted to GSSG by glutathione peroxidase, whereas GSSG is converted to GSH by glutathione reductase (Knollema et al., 1996; Lubos et al., 2011). The GSH-GSSG cycle is involved in intracellular processses including (1) conversion of hydrogen peroxide (H_2_O_2_) to water and oxygen, (2) maintaining cell redox/antioxidant status, (3) detoxification of xenobiotic agents, (4) maintaining bioavailable pools of L-cysteine, (5) production of iron-sulfur (Fe–S) cluster proteins, and (6) synthesis and storage of preformed pools of S-nitrosothiols (Dringen et al., 2000; Ghezzi, 2005; Franco et al., 2007; Seth and Stamler, 2011; Ghezzi and Chan, 2017; Seckler et al., 2017; Seckler et al., 2020; Seckler et al., 2022). Depleted levels of GSH trigger ROS generation implicated in cell death causing neurological diseases like Alzheimer’s disease, Parkinson’s disease and multiple sclerosis (Lovell et al., 1995; Pearce et al., 1997; Choi et al., 2011; Saharan and Mandal, 2014). A key finding of the present study is that D-CYSee normalized the levels of GSH and GSH/GSSG ratio in SH-SY5Y cells. Whether D-CYSee enhances GSH levels by directly interfering with morphine/opioid-receptor-initiated changes in the activity of enzymes regulating intracellular levels of GSH (e.g., glutathione peroxidase and glutathione reductase) remains to be determined. Although we have established that D-CYSee and related L,D-thiolesters do not directly antagonize opioid-receptors in rats (Gaston et al., 2021; Jenkins et al., 2021; Getsy et al., 2022a; Getsy et al., 2022b; Getsy et al., 2022c; Getsy et al., 2022d; Lewis et al., 2022), it is possible that the application of D-CYSee under the present experimental conditions (overnight incubation) causes a downregulation of plasma membrane opioid receptors in the SH-SY5Y cells *per se* rather than overcoming opioid-receptor signaling events.

The present study also found that overnight incubation with morphine caused substantial damage to the SH-SY5Y cells as evidenced by the deleterious changes in mitochondrial membrane potential. The mechanisms by which morphine causes injury to mitochondria are multi-factorial and involve, oxidative stress (e.g., increased levels of reactive oxygen species); lipid peroxidation; upregulation of activity of caspase-3 and caspase-9, Drp1 and Mfn2; generation of neuroinflammatory cytokines (e.g., IL-1β, TNF-α, IL-6); raising intracellular Ca^2+^ to neurotoxic levels (Feng et al., 2013; MacVicar et al., 2015; Kasala et al., 2020; Osmanlıoğlu et al., 2020); and generation of the powerful oxidant/nitrating agent, peroxynitrite (Muscoli et al., 2007). The morphine-induced damage to SH-SY5Y cells may represent early stages of cell death, since morphine induces Beclin 1- and ATG5-dependent autophagy in these cells (Zhao et al., 2010). An exciting finding of the present study was that D-CYSee prevented morphine-induced damage to mitochondria in human SH-SY5Y cells. It is likely that the antioxidant ability of D-CYSee (Getsy et al., 2022e; Getsy et al., 2022f) is involved in this effect since other antioxidants, including curcumin (Motaghinejad et al., 2015), recombinant human growth hormone (Nylander et al., 2016), and the mitochondrial-targeted antioxidant, melatonin (Feng et al., 2013), protect cells from morphine-induced damage. It should be noted that the kappa opioid receptor agonist Salvinorin A reduced the levels of reactive oxygen species, thereby protecting membrane potential and morphology of mitochondria by upregulating the phosphorylation levels of AMPK, and increasing Mfn2 expression (Dong et al., 2019). Moreover, Luo et al. (2013) reported that morphine induced cell damage via the mitochondria-mediated apoptosis pathway by processes involving the activation of caspases-3 and caspases-9, were attenuated by pre-treatment with geranylgeranylacetone, a pharmacological inducer of Trx-1 and Hsp70. All of the above findings are consistent with evidence that the opioid receptor agonist, Tramadol, causes (a) oxidative damage to proteins in mitochondria of SH-SY5Y cells (Faria et al., 2016), and (b) oxidative damage (ROS overproduction) in mitochondria, and via deleterious changes in activity of Complex II (succinate dehydrogenase), in addition to membrane permeability, transition pore disorder, collapse of mitochondrial membrane potential and mitochondria swelling (Mohammadnejad et al., 2021). Furthermore, Samadi et al. (2021) found that caffeine, a nonselective antagonist of adenosine receptors, markedly diminished the ability of Tramadol to increase oxidative stress biomarkers, such as reactive oxygen species, protein carbonyl content, and lipid peroxidation, and to decrease GSH content in brain mitochondria. We found that D-CYSee prevented morphine-induced decreases in global DNA methylation in human SH-SY5Y cells perhaps by enhancing S-adenosylmethionine (SAM)-dependent changes in DNA methyltransferase (DNMT)- and histone methyltransferase (HMT)-dependent processes that drive the methylation status of DNA and histones (Figure 1). Our study also confirms the findings of Trivedi et al. (2014) that morphine decreased 5-mC levels in the LINE-1 gene and increased LINE-1 RNA in SH-SY5Y cells. The LINE-1 gene is a retrotransposon. These genes are relics of viral like sequences that infect the genome and become stably inserted and inherited (McClung et al., 2005). Regulatory sequences surrounding retrotransposons are typically hyper-methylated and so these genes, while they have been evolutionarily maintained in the genome, are silenced. The significance of opioid-induced changes in expression of these genes, and their potential contribution to opioid-induced changes in physiological status, is not clear at present, but it is evident that D-CYSee prevents these morphine-induced changes from happening. As such, it is evident that D-CYSee can maintain redox homeostasis and protect mitochondria from opioid-induced toxicity by mechanisms that may involve the generation of GSH from GSSG (Figure 3). That presence of D-cysteine, and the parallel presence of the enzyme, serine racemase, which interconverts D-cysteine and L-cysteine, as well as D- and L-serine (hence the name serine racemase), implies that another possible mechanistic pathway is D-CYSee - > D-cysteine - > L-cysteine (Semenza et al., 2021).

Betaine is synthesized in mitochondria from choline via choline dehydrogenase (Wang et al., 2006; Ohnishi et al., 2019; Arumugam et al., 2021) and ingested in the diet (He et al., 2012). Betaine is actively transported into cells by organic osmolyte transporter betaine/γ-aminobutyric acid (GABA) transporter BGT1 (SLC6A12) (Lehre et al., 2011; Munoz et al., 2012; Zhou and Danbolt, 2013) that is a member of the Na^+^- and Cl^−^-dependent neurotransmitter transporter gene family (solute carrier family 6, neurotransmitter, sodium symporter transporter family) with a homology to GABA transporters GAT1 (SLC6A1), GAT2 (SLC6A13) and GAT3 (SLC6A11) (Gerile et al., 2012; Lie et al., 2020; Bhatt et al., 2023). BGT1 activity and expression is regulated by AMP-activated kinase (Munoz et al., 2012), and plays a role in controlling brain excitability (Kempson et al., 2014). Betaine has several biological activities, including (a) anti-oxidative and anti-inflammatory activity (Zhao et al., 2010); (b) provision of the methyl donor, S-adenosylmethionine (Lever and Slow, 2010); (c) key regulator of cellular osmotic status (Chen and Murata, 2008; Knight et al., 2017); and (d) mitigation of the pathologies associated with elevated homocysteine levels (Kumar et al., 2016) (see Supplementary Figure S1 from Ohnishi et al., 2019). The actions of betaine involve the accelerated turnover of the methionine-homocysteine cycle (i.e., one-carbon metabolism/folate cycle), where betaine is a substrate in the betaine-homocysteine S-methyltransferase (BHMT) reaction, which converts homocysteine to the essential reducing compound, methionine (Ueland et al., 2005; Ueland, 2011). Deficits in brain betaine levels may contribute to cellular osmotic perturbation (Knight et al., 2017; Chen and Murata, 2008; Kempson and Montrose, 2004), which is reported to inhibit methionine uptake, inhibit protein synthesis, and affect mRNA translation, by dysregulation of phosphorylation and mTOR signaling cascades (Uesono and Toh, 2002; Patel et al., 2002). Betaine readily penetrates the blood-brain barrier and is well tolerated with few adverse effects (Aramburu et al., 2014). Our pivotal findings in SH-SY5Y cells were that betaine prevented morphine-induced decreases in GSH concentrations (although somewhat less effectively than D-CYSee), restored mitochondrial membrane potential during morphine treatment, prevented morphine-induced decreases in global DNA methylation, and increased expression of LINE-1 RNA. These key effects of betaine are possibly driven by betaine homocysteine methyl-transferase (BHMT)- and methionine adenosyltransferase (MAT)-driven production of SAM-dependent methylation of DNA and histones (Figure 1) (García-Giménez et al., 2017; Wang et al., 1997). As such, it is evident that betaine, like D-CYSee, is able to maintain redox homeostasis and protect mitochondria from opioid-induced toxicity via potential mechanisms that include the generation of methionine from homocysteine (Figure 3).

The present study also demonstrates that the bolus injection of NLX elicited a pronounced withdrawal syndrome in rats that received the escalating morphine injection dose regime plus co-injections of vehicle. The behavioral phenomena consistant with the rats having become dependent on morphine consisted of jumping, wet dog-like shakes, rearing, fore-paw licking (FPL), circling, full body writhing, and sneezing. These, and the recorded falls in body temperature and body weight, are common features of the NLX-induced withdrawal syndrome in morphine-dependent rats (Laska and Fennessy, 1976; Hutchinson et al., 2007; Morgan and Christie, 2011; Nielsen and Kreek, 2012). The withdrawal responses elicited by the injection of NLX in rats that had received co-injections of morphine plus vehicle were qualitatively and quantitatively similar in the rats that had received co-injections of morphine plus D-cysteine. D-cysteine is a naturally occurring amino acid (Kiriyama and Nochi, 2016; Seckler and Lewis, 2020) that can readily be detected in mouse brain (Semenza et al., 2021), although it cannot be readily detected in the brains of other species (Mangas et al., 2007; Seckler and Lewis, 2020). Nonetheless, there is a variety of uptake processes for exogenously administered D-cysteine (Glazenburg et al., 1984; Pisoni et al., 1990; Huang et al., 1998; Simmons-Willis et al., 2002; Erdogan et al., 2021), which has a multiplicity of neurological actions (Seckler and Lewis, 2020). In addition to expected redox effects (Hobbs et al., 1998; Homma et al., 2022), D-cysteine generates intracellular hydrogen sulfide via the D-amino acid oxidase/3-mercaptopyruvate sulfurtransferase pathway (Kimura, 2013; Souza et al., 2017). Accordingly, the lack of effect of the dose of D-cysteine used in this study (250 μmol/kg, = 30.3 mg/kg per each injection) may be because it does not enter brain cells involved in establishing physical dependence to morphine in sufficient quantities to exert meaningful cell-signaling events that can countermand the processes underlying the development of dependence. A major finding of the present study was that the injection of NLX elicited a relatively minor withdrawal syndrome in rats that had received co-injections of morphine and D-CYSee. This finding that the D-thiol ester prevents the development of physical dependence to morphine, is consistent with our novel evidence described above that D-CYSee prevents redox and epigenetic signatures of opioid dependence in human SH-SH5Y neuroblastoma cells treated overnight with morphine and D-CYSee. These findings are supported strongly by another recent finding that D-CYSee prevents fentanyl-induced reward seeking in male and female rats (Knauss et al., 2023).

Chronic opioid administration causes impairment of mitochondrial function (e.g., Bcl-2, Bcl-x_L_, Bad, and Bax apoptotic pathways) within the brain (Tapia-Arizmendi et al., 1987; García-Estrada et al., 1988; Tramullas et al., 2007; Bekheet et al., 2010) by mechanisms involving the production of reactive oxygen-nitrogen species, such as peroxynitrite (Muscoli et al., 2007; Doyle et al., 2009), which directly contributes to impairment of spatial learning and memory (Tramullas et al., 2008). In addition, Luo et al. (2022) reported that heroin addiction in rats markedly diminishes expression of mitochondrial enzymes, such as cytochrome c oxidase IV and ATP synthase subunit beta. Moreover, Gowen et al. (2023) recently reported that neonatal opioid exposure causes neuroinflammation, and adversely affects the synaptic proteome, mitochondrial function, and behavior in juvenile rats. With respect to the therapeutic efficacy of D-CYSee, we do not have full understanding of the mechanisms by which this D-thiol ester ameliorates the development of physical dependence to morphine *in vivo*. On the basis of our findings that D-CYSee reverses the effects of morphine on GSH/GSSG ratio in SH-SY5Y cells, it is likely that the antioxidant/reducing properties of D-CYSee allows direct modulation of intracellular redox status (e.g., reduction of L-cystine to L-cysteine and conversion of Fe^3+^ to Fe^2+^ in heme proteins), and activity of membrane proteins, such as Kv_1.2_ K^+^-channels (Baronas et al., 2017) and functional intracellular proteins (Bogeski et al., 2011; Bogeski and Niemeyer, 2014; O-Uchi et al., 2014; Gamper and Ooi, 2015; Gao et al., 2017; García et al., 2018). Indeed, antioxidants, such as L-NAC, can diminish the development of tolerance and physical dependence to morphine (Abdel-Zaher et al., 2013a; Abdel-Zaher et al., 2013b; Yun et al., 2015; Yun et al., 2017; Yayeh et al., 2016), whereas co-administration of the antioxidants ascorbic acid, uric acid, glutathione, quercetin, and resveratrol, diminish development of oxidative stress and NLX-precipitated withdrawal syndrome in heroin-treated mice (Pan et al., 2005; Xu et al., 2006). Other mechanisms may involve (1) formation of thiol adducts, such as D-glucose:D-cysteine (Wróbel et al., 1997; Szwergold, 2006; Li et al., 2015) and mixed disulfides Wilcken and Gupta, 1979; Lash and Jones, 1985; Turell L et al., 2013) in blood, (2) conversion of D-CYSee to D-cysteine by membrane associated esterases (Butterworth et al., 1993; Nishida et al., 1996), which enter into intracellular signaling pathways, such as those generating hydrogen sulfide, by sequential actions of D-aminoacid oxidase and 3-mercaptopyruvate sulfurtransferase (Kimura, 2014; Kimura, 2014; Kimura, 2017; Beltowski, 2019), and (3) nitric oxide synthase-dependent generation of S-nitroso-D-cysteine ethyl ester and S-nitroso-D-cysteine that may behave like the endogenous S-nitrosothiol, S-nitroso-L-cysteine (Myers et al., 1990; Bates et al., 1991; Seckler et al., 2017; Seckler et al., 2020), which regulates intracellular signaling pathways (Lipton et al., 1993; Stamler, 1995; Foster et al., 2009; Seth and Stamler, 2011; Gaston et al., 2020), including those controlling cardiovascular and ventilatory functions (Davisson et al., 1996; Davisson et al., 1997; Ohta et al., 1997; Lipton et al., 2001; Gaston et al., 2006; Lewis et al., 2006; Gaston et al., 2020) and those that reverse OIRD (Getsy et al., 2022a; Getsy et al., 2022b).

These and other mechanisms may interact with brain signaling pathways involved in the acquisition of physical dependence to opioids and expression of NLX-precipitated withdrawal, including pathways involving N-methyl D-aspartate (NMDA) glutamatergic receptors (Buccafusco et al., 1995; Herman et al., 1995; Rasmussen, 1995; Noda and Nabeshima, 2004; Glass, 2011; Fluyau et al., 2020), muscarinic receptors (Marshall and Buccafusco, 1985; Holland et al., 1993), corticotropin releasing factor (CRF) receptor CRF1 (García-Carmona et al., 2015), tachykinin receptors (Michaud and Couture, 2003), voltage-gated Ca^2+^-channels (Tokuyama et al., 1995; Dogrul et al., 2002; Esmaeili-Mahani et al., 2008; Alboghobeish et al., 2019), adenylyl cyclase super-activation and phosphorylation of opioid receptor (Avidor-Reiss et al., 1996; Avidor-Reiss et al., 1997; Wang et al., 1999; Eckhardt et al., 2000), oxidative stress (Mori et al., 2007; Abdel-Zaher et al., 2013a; Abdel-Zaher et al., 2013b; Mansouri et al., 2020; Ward et al., 2020; Houshmand et al., 2021), and the nitric oxide-cGMP signaling pathway (Adams et al., 1993; Cappendijk et al., 1993; Majeed et al., 1994; Leza et al., 1995; Leza et al., 1996; London et al., 1995; Vaupel et al., 1995a; Vaupel et al., 1995b; Dambisya and Lee, 1996; Bhatt and Kumar, 2015; Tsakova et al., 2015; Sackner et al., 2019; Gledhill and Babey, 2021). Since D-CYSee markedly attenuated all NLX-precipitated behavioral (except for sneezes) and physical (hypothermia and body weight loss) phenomena, it is possible that D-CYSee modulates fundamental intracellular processes that are critical to the development of physical dependence to morphine in male Sprague Dawley rats. The above discussion is directly relevant to potential mechanisms by which betaine diminished the development of dependence to morphine. An outstanding difference between the effects of betaine and D-CYSee was that unlike D-CYSee, betaine also diminished the occurrence of NLX-precipitated sneezing. Sneezing is a common feature of opioid withdrawal in humans (Ostrea et al., 1975; Specker et al., 1998; Gaalema et al., 2012; Lofwall et al., 2013) and experimental animals (Hendrie, 1985; Liu et al., 2007; Singh et al., 2015). There is considerable information about the neural mechanisms driving sneezing (Batsel and Lines, 1975; Undem et al., 2000; Li et al., 2021; Ramirez et al., 2022), and it is now evident that the cellular events initiated by betaine can be added to these potential mechanisms.

## Study limitations

There are several limitations that need to be described. With regards to the studies in the human SH-SH5Y neuroblastoma cells, it is vital to perform studies with longer-term application of morphine, and establish fuller dose-response curves to D-CYSee and betaine. Despite the strength of findings in SH-SH5Y cells, it is imperative to determine how D-CYSee and betaine affect chronic morphine-induced changes in redox (e.g., GSH, GSSG) and epigenetic signatures of physical dependence in brain regions involved in development of physical dependence to opioids, such as medial prefrontal cortex (MPFC), striatum and hippocampus (Deslandes et al., 2002; Gardner, 2011; Koob and Volkow, 2016; Volkow et al., 2019; Koob, 2020; Sakloth et al., 2020). In addition, future studies must establish whether D-CYSee and betaine can overcome physical dependence to fentanyl, since this synthetic opioid has an ever-increasing role in the current opioid crisis (Arendt, 2021; Deo et al., 2021). Another important limitation of our studies is the lack of data about the efficacy of D-CYSee and betaine in preventing the adverse biochemical actions of morphine in female cells, and reversing physical dependence in female rats. This is essential since (a) there are numerous sex-specific differences in opioid receptor signaling (Bryant et al., 2006; Hosseini et al., 2011), (b) opioids have often different responses (e.g., ventilation, analgesia) in females compared to males (Dahan et al., 1998; Sarton et al., 1998; Bodnar and Kest, 2010), (c) there are major sex-dependent differences in development of opioid tolerance/hyperalgesia, and expression of withdrawal responses (Bodnar and Kest, 2010) and (d) there are several major sex differences in the efficacy of treatments for OUD (Huhn et al., 2019; Davis et al., 2021; Knouse and Briand, 2021).

## Conclusion

This study provides evidence that application of D-CYSee or betaine prevents the expression of the epigenetic signatures associated with morphine physical dependence/addiction in human SH-SH5Y neuroblastoma cells, and lessens the development of physical dependence to morphine. Our previous studies have demonstrated that the sulfur atom of D-CYSee is vital to the activity of the D-thiol ester (Getsy et al., 2022e; Getsy et al., 2022f), and defining thiol/S-nitrosothiol-dependent signaling pathways (Belcastro et al., 2017; Stomberski et al., 2019) will add greatly to our understanding of how opioids induce dependence, and the mechanisms by which D-thiol esters and D-cysteine (Bonifácio et al., 2021) exert their effects. Trivedi et al. (2014a, 2014b) provided compelling evidence that morphine may cause dependence/addiction by blocking the entry of L-cysteine into neurons by inhibition of the EAA3/EAAC1 transporter, thereby reducing L-cysteine-dependent cell signaling pathways (Yamaguchi and Hosokawa, 1987; Rossi et al., 2009; Stipanuk et al., 2009; Stipanuk et al., 2011). The findings that betaine and D-CYSee markedly reduced the majority of NLX-precipitated withdrawal phenomena suggests that the loss of L-cysteine entry into cells plays a key role in establishing physical dependence to morphine. Additionally, our findings show that betaine and D-CYSee somehow overcome the loss of endogenous L-cysteine in intracellular signaling processes that allow for the development of morphine dependence and addiction. The present findings add to our increasing knowledge about the efficacy of L,D-thiolesters, Tempol, and S-nitroso-L-cysteine in overcoming the adverse action of opioids (Baby et al., 2021a; Baby et al., 2021b; Gaston et al., 2021 Getsy et al., 2022a; Getsy et al., 2022b; Getsy et al., 2022c; Getsy et al., 2022d; Getsy et al., 2022e; Getsy et al., 2022f; Lewis et al., 2022).

## Data Availability

The raw data supporting the conclusions of this article will be made available by the authors, without undue reservation.
